# Origin, differentiation and functional ultrastructure of egg envelopes in the cestode *Echinococcus multilocularis* Leuckart, 1863 (Cyclophyllidea: Taeniidae)

**DOI:** 10.1007/s00436-017-5479-x

**Published:** 2017-06-07

**Authors:** Zdzisław Świderski, Jordi Miquel, Samira Azzouz-Maache, Anne-Françoise Pétavy

**Affiliations:** 1 Witold Stefański Institute of Parasitology, Polish Academy of Sciences, 51/55 Twarda Street, 00-818 Warszawa, Poland; 2 Secció de Parasitologia, Departament de Biologia, Sanitat i Medi Ambient, Facultat de Farmàcia i Ciències de l’Alimentació, Universitat de Barcelona, Av. Joan XXIII, sn, 08028 Barcelona, Spain; 3 Institut de Recerca de la Biodiversitat (IRBio), Facultat de Biologia, Universitat de Barcelona, Av. Diagonal, 645, 08028 Barcelona, Spain; 4 Laboratoire de Parasitologie et Mycologie Médicale, Faculté de Pharmacie, Université Claude Bernard-Lyon 1, 8 Av. Rockefeller, 69373 Lyon, Cedex 08 France

**Keywords:** *Echinococcus multilocularis*, Cestoda, Taeniidae, Egg envelopes, Envelopes origin and differentiation, Functional ultrastructure

## Abstract

The origin, differentiation and functional ultrastructure of oncospheral or egg envelopes in *Echinococcus multilocularis* Leuckart, 1863 were studied by transmission electron microscopy (TEM) and cytochemistry. The purpose of our study is to describe the formation of the four primary embryonic envelopes, namely vitelline capsule, outer envelope, inner envelope and oncospheral membrane, and their transformation into the oncospheral or egg envelopes surrounding the mature hexacanth. This transformation takes place in the preoncospheral phase of embryonic development. The vitelline capsule and oncospheral membrane are thin membranes, while the outer and inner envelopes are thick cytoplasmic layers formed by two specific types of blastomeres: the outer envelope by cytoplasmic fusion of two macromeres and the inner envelope by cytoplasmic fusion of three mesomeres. Both outer and inner envelopes are therefore cellular in origin and syncytial in nature. During the advanced phase of embryonic development, the outer and inner envelopes undergo great modifications. The outer envelope remains as a metabolically active layer involved in the storage of glycogen and lipids for the final stages of egg development and survival. The inner envelope is the most important protective layer because of its thick layer of embryophoric blocks that assures oncospheral protection and survival. This embryophore is the principal layer of mature eggs, affording physical and physiological protection for the differentiated embryo or oncosphere, since the outer envelope is stripped from the egg before it is liberated. The embryophore is very thick and impermeable, consisting of polygonal blocks of an inert keratin-like protein held together by a cementing substance. The embryophore therefore assures extreme resistance of eggs, enabling them to withstand a wide range of environmental temperatures and physicochemical conditions.

## Introduction

In the family Taeniidae, the genus *Echinococcus* includes species of great medical and veterinary importance, causing the important zoonotic infections cystic and alveolar echinococcosis. *Echinococcus multilocularis* is the zoonotic agent of human alveolar echinococcosis or alveolar hydatid disease. Its indirect life cycle includes wild canids and also dogs as definitive hosts harbouring the adult tapeworm, whereas some micromammals act as intermediate hosts harbouring the larval stage. Human infections with the metacestode of *E. multilocularis* result in alveolar hydatid disease which is still very common in different countries.

Formation of embryonic envelopes and their transformation into oncospheral or egg envelopes, as well as their number and terminology, are the most confusing topics of cestode embryogenesis (Rybicka 1965, 1966; Sakamoto 1981; Świderski 2008). Comparative study has been very difficult largely because of the lack of uniform terminology used by various authors. As early as 1966, Rybicka was trying to review and compare different terms used by various authors for the same oncospheral and egg envelopes (see Table 1 of Rybicka 1966). However, very recently, the problem of standardised terminology of embryonic envelopes of tapeworm was reviewed and updated by Conn and Świderski (2008) and is used in the present study.

The oncospheral or egg envelopes play an important role in protection, nutrition and metabolism of the infective oncospheres of cestodes (Rybicka 1965, 1966; Świderski 1983, 1988, 2008). They exhibit a large variety of modes of differentiation and their ultrastructure as related to the wide range of different cestode life cycles and their adaptations to different hosts and environmental conditions (Pence 1967, 1970; Nieland 1968; Świderski 1968, 1972, 1975, 1981, 2008; Świderski and Eckert 1977; Fairweather and Threadgold 1981; Conn 1988, 2000; Chomicz et al. 1995; Tkach and Świderski 1997, 1998; Świderski et al. 2000, 2001; Chomicz and Świderski 2004; Młocicki et al. 2005).

The scarcity of ultrastructural data on taeniid eggs may be partly explained by several serious disadvantages they present for electron microscope examination: a very thick, dense, rigid and impermeable embryophore composed of a thick layer of keratin-like protein blocks. These technical difficulties have impeded getting taeniid eggs properly fixed and infiltrated with embedding media to allow for cutting of the thick layer of keratinised embryophores (Świderski 1982, 1983, 1988, 1994) and dense, keratinised oncospheral hooks (Świderski et al. 2016a).

The purpose of the present study is to describe the origin, differentiation and functional ultrastructure of the oncospheral envelopes surrounding the eggs of *Echinococcus multilocularis* and to compare the results with published data on taeniids and other cyclophyllideans. However, the so-called hook region membrane that covers only one pole of the mature oncosphere and is directly attached to the oncosphere surface will be subject of a separated publication.

## Materials and methods

### Materials

Live specimens of *E. multilocularis* were isolated from the intestine of a naturally infected red fox (*Vulpes vulpes* L.) from La Roche sur Foron (France) captured in June 2014.

### TEM preparation of samples

Adult tapeworms were immediately rinsed with a 0.9% NaCl solution. Later, they were fixed in cold (4 °C) 2.5% glutaraldehyde in a 0.1 M sodium cacodylate buffer at pH 7.4 for a minimum of 2 h, rinsed in 0.1 M sodium cacodylate buffer at pH 7.4, post-fixed in cold (4 °C) 1% osmium tetroxide with 0.9% potassium ferricyanide in the same buffer for 1 h, rinsed in Milli-Q water (Millipore Gradient A10), dehydrated in an ethanol series and propylene oxide, embedded in Spurr’s resin and polymerised at 60 °C for 72 h.

Egg development was followed by selecting proglottids in different stages of maturation. Ultrathin sections (60–90 nm thick) of these mature and gravid proglottids at different levels were obtained with a Reichert-Jung Ultracut E ultramicrotome. Sections were placed on 200-μm mesh copper grids and double-stained with uranyl acetate and lead citrate according to the Reynolds (1963) methodology. The grids were examined in a JEOL 1010 transmission electron microscope (Jeol, Japan) operated at 80 kV, in the “Centres Científics i Tecnològics” of the University of Barcelona (CCiTUB).

### Freeze substitution and infiltration with Lowicryl resin

Some specimens were fixed in cold (4 °C) 4% paraformaldehyde + 0.1% glutaraldehyde in a 0.1 M sodium cacodylate buffer at pH 7.4 for a 4 to 5 h and then conserved in cold (4 °C) 2% paraformaldehyde in the same buffer. Samples were rinsed in a 0.15 M glycine in a 0.1 M sodium cacodylate buffer at pH 7.4, cryoprotected by crescent concentrations (10, 20 and 30%) of glycerol in the same buffer and then cryofixed in liquid propane.

Samples were freeze-substituted for 3 days at −90 °C in anhydrous acetone containing 0.5% uranyl acetate. Then, they were warmed up to −50 °C, at 5 °C/h (EM AFS2, Leica, Vienna, Austria). After several acetone rinses, samples were infiltrated with Lowicryl HM20 resin during 4 days. Samples were polymerised under UV light at −50 °C for 24 h, during the warming up at a rate 5 °C/h until 22 °C and 48 h at 22 °C.

Ultrathin sections were picked up on Formvar-coated nickel grids, double-stained with uranyl acetate and lead citrate and examined in a JEOL 1010 TEM operated at 80 kV, in the CCiTUB.

### Cytochemistry

The periodic acid-thiocarbohydrazide-silver proteinate (PA-TCH-SP) technique of Thiéry (1967) was applied to determine the cytochemical localisation of glycogen at the ultrastructural level. Thus, ultrathin sections collected on gold grids were treated as follows: 30 min in 10% PA, rinsed in Milli-Q water; 24 h in TCH, rinsed in acetic solutions and Milli-Q water; and 30 min in 1% SP in the dark and rinsed in Milli-Q water. Gold grids were also examined in a JEOL 1010 TEM operated at an accelerating voltage of 80 kV, in the CCiTUB.

## Results

### Origin and differentiation of egg envelopes

**Fig. 1:**
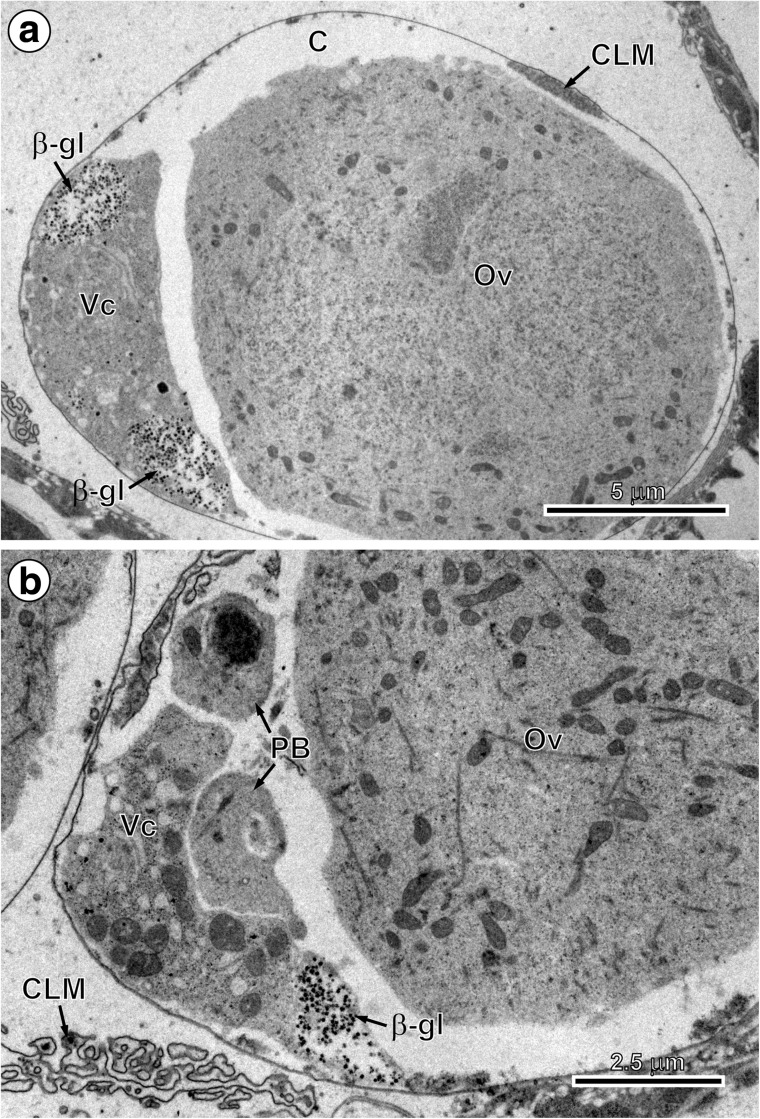
The early stages of embryonic development in *Echinococcus multilocularis*. **a** Fertilised oocyte or ovum and adjacent vitellocyte both surrounded by a newly formed vitelline capsule. Note capsule labyrinth membrane complex on the upper surface of the newly formed vitelline capsule and numerous β-glycogen granules in the cytoplasm of vitellocyte. **b** Part of an early embryo composed of oocyte, remnants of the vitelline cell with β-glycogen particles and two polar bodies. Note infolded labyrinth of capsular membrane in the left lower corner of the micrograph. *β-gl* β-glycogen, *C* vitelline capsule, *CLM* capsule labyrinth membrane, *Ov* oocyte, *PB* polar body, *Vc* vitellocyte

The first embryonic envelope, the vitelline capsule, is a thin proteinaceous layer that forms from coalescence of material released by exocytosis from a single vitellocyte. This occurs after oocyte fertilisation and during passage of the fertilised ova and vitellocytes through the ootype that is surrounded by Mehlis’ gland cells. It was observed at this stage that the vitelline material from the vitellocyte cytoplasm is transformed simultaneously into a labyrinth-like reservoir membrane which appears on the surface of the vitelline cell. The labyrinth unrolls, flattens and becomes smooth, with the exception of certain regions still remaining for a variable period in the labyrinthine state (Fig. 1a, b). It is referred to as a “labyrinth-like membrane” by Świderski et al. (1970) or capsule “reticular mass” by Conn (1988). The role of Mehlis’ gland secretion on the release of vitelline material from the vitellocytes appears evident.

**Fig. 2:**
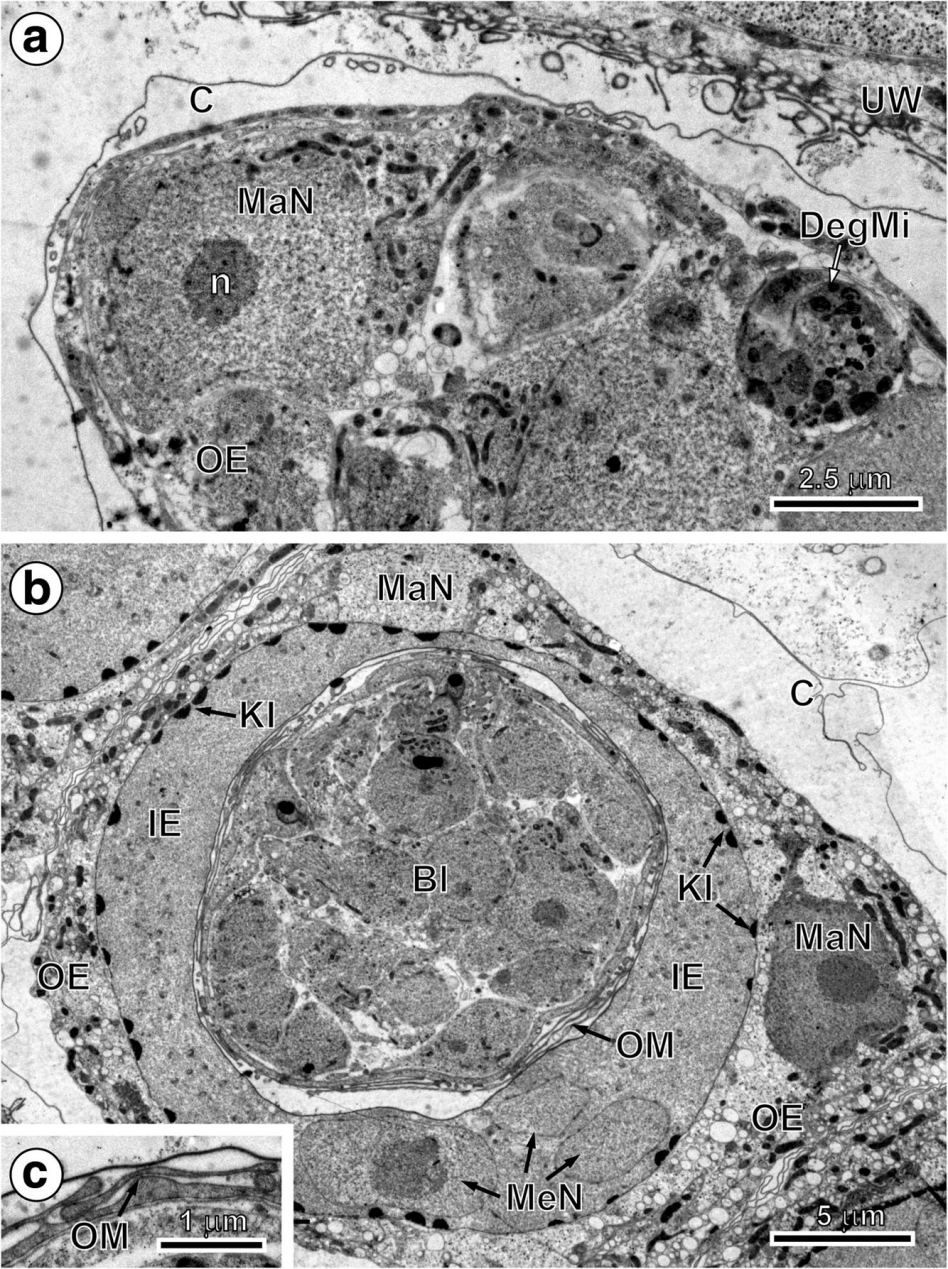
Initial stages of the outer and inner envelope formation. **a** Part of an early embryo adjacent to uterine wall. Note (1) already formed membraneous vitelline capsule surrounding an early embryo composed of several blastomeres of different sizes and (2) a large macromere, situated under the vitelline capsule at the periphery of other blastomeres, which contains predominant nucleus with spherical electron-dense nucleolus, which takes part in the outer envelope formation. **b** Preoncospheral phase in more advanced stage of embryonic development. Four primary embryonic envelopes (vitelline capsule, outer envelope, inner envelope and oncospheral membrane) are clearly visible. In the outer envelope, note large nucleus of macromeres which predominant nucleolus, numerous elongated mitochondria and higher concentration of free ribosomes. In the inner envelope, note three nuclei of mesomeres, the fusion of which forms the cytoplasm of the syncytial layer. **c** Detail of the oncospheral membrane. *Bl* blastomere, *C* vitelline capsule, *DegMi* degenerating micromere, *IE* inner envelope, *KI* keratin-like protein islands, *MaN macromere* nucleus, *MeN* mesomere nucleus, *n* nucleolus, *OE* outer envelope, *OM* oncospheral membrane, *UW* uterine wall

**Fig. 3:**
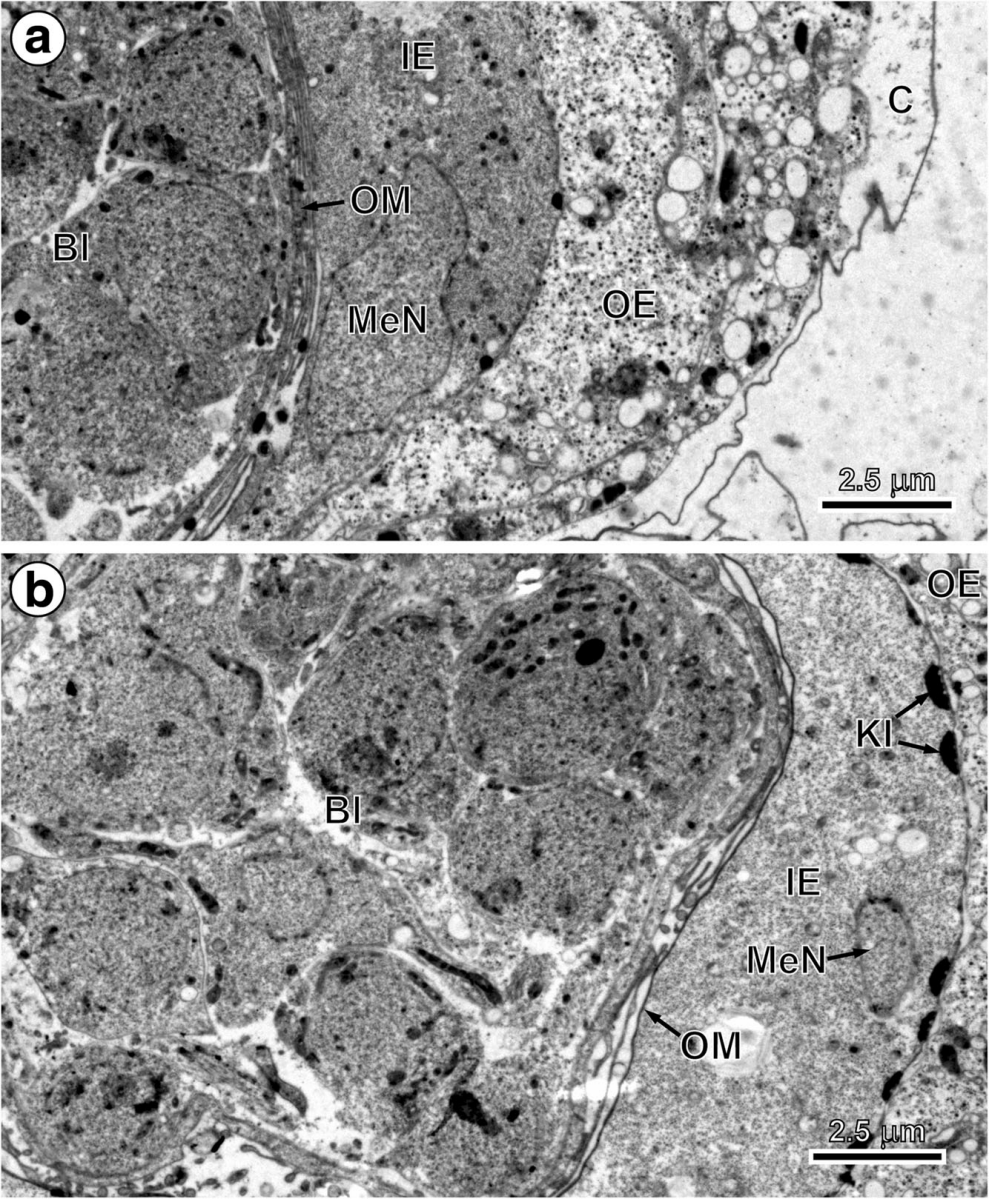
Ultrastructure of embryos in advanced preoncospheral phase of development. **a** Part of an embryo surrounded by four embryonic envelopes, namely vitelline capsule, outer envelope, inner envelope with nucleus of mesomere and oncospheral membrane. **b** Part of an embryo composed of numerous blastomeres. Note the initial stage of embryophoric block formation composed of small electron-dense islands of keratin-like protein situated under the outer membrane of the inner envelope which shows the nucleus of mesomere participating in its formation. *Bl* blastomere, *C* vitelline capsule, *IE* inner envelope, *KI* keratin-like protein islands, *MeN* mesomere nucleus, *OE* outer envelope, *OM* oncospheral membrane

**Fig. 4:**
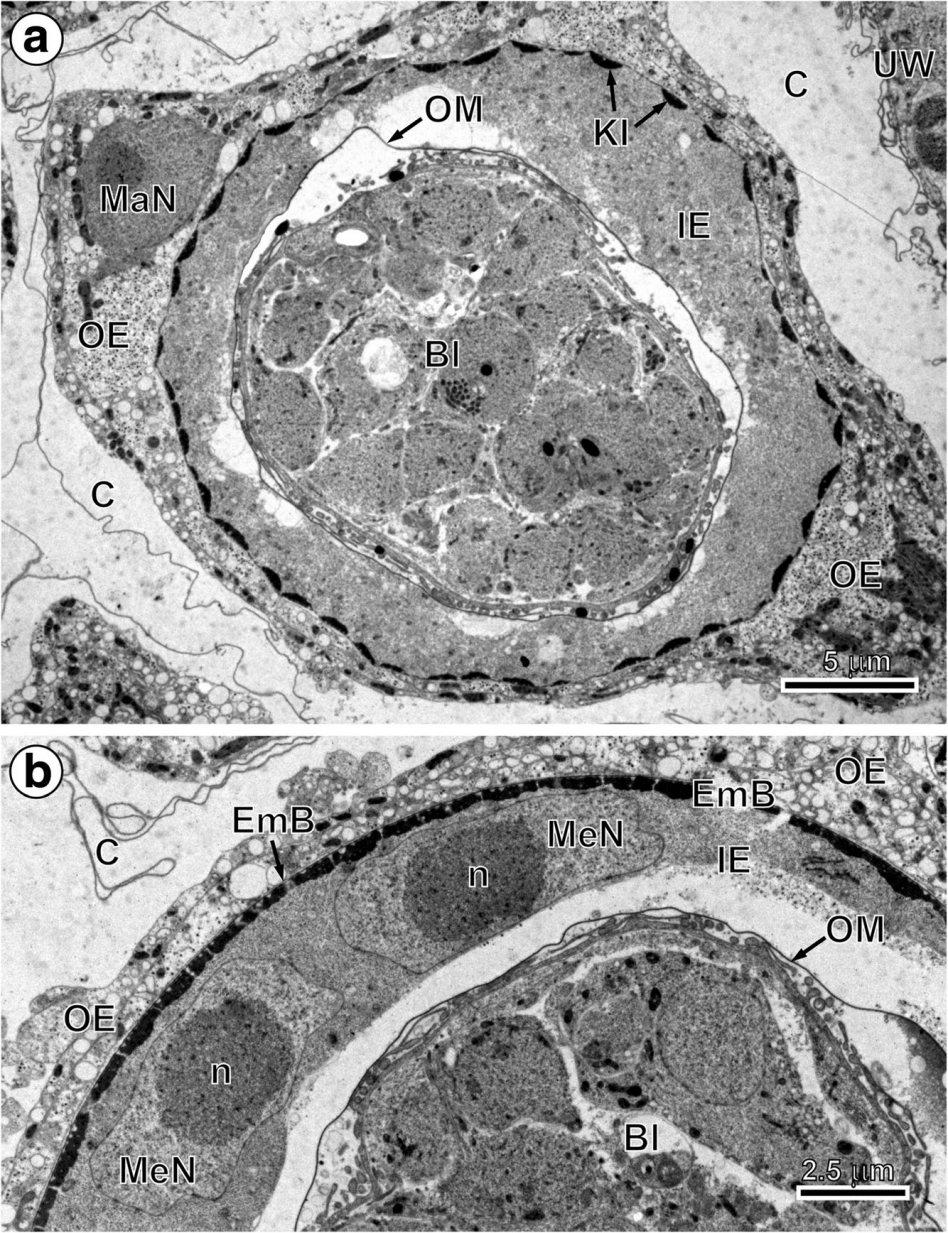
Ultrastructure of *Echinococcus multilocularis* eggs in the middle and more advanced preoncospheral phase of the embryonic development. **a** Median cross section through the entire egg in the middle phase of preoncospheral development. Note the (1) presence of four embryonic envelopes (vitelline capsule, outer envelope, inner envelope and oncospheral membrane), undergoing differentiation into oncospheral or egg envelopes, (2) presence of a large nucleus of macromere in the outer envelope, (3) presence of a discontinuous layer of flat embryophoric islands of keratin-like protein adjacent to the outer membrane of the inner envelope and (4) evident detachment of the oncospheral membrane from the inner surface of the inner envelope which participated in its formation. **b** Part of the embryo showing a thin layer of embryophoric blocks and two large flattened mesomere nuclei with prominent electron-dense nucleoli situated in the deeper granular sub-layer of the inner envelope. *Bl* blastomere, *C* vitelline capsule, *EmB* embryophoric blocks of keratin-like protein, *IE* inner envelope, *KI* keratin-like protein islands, *MaN* macromere nucleus, *MeN* mesomere nucleus, *n* nucleolus, *OE* outer envelope, *OM* oncospheral membrane, *UW* uterine wall

In the early and advanced preoncospheral phase of development of the taeniid cestode *E. multilocularis*, four primary embryonic envelopes are formed: vitelline capsule, outer envelope, inner envelope and oncospheral membrane (Figs. 2b, c, 3a, b and 4a, b). Two of them, the vitelline capsule and oncospheral membrane, are simply thin membranes (Figs. 2b, c, 3a and 4a, b), while the outer and inner envelopes are thick cytoplasmic layers formed by two specific types of blastomeres: the outer envelope is formed by cytoplasmic fusion of two macromeres and the inner envelope by a cytoplasmic fusion of three mesomeres (Figs. 2b, 3a, b and 4a, b). Both the outer and inner envelopes of *E. multilocularis* are therefore cellular in origin and syncytial in nature.

**Fig. 5:**
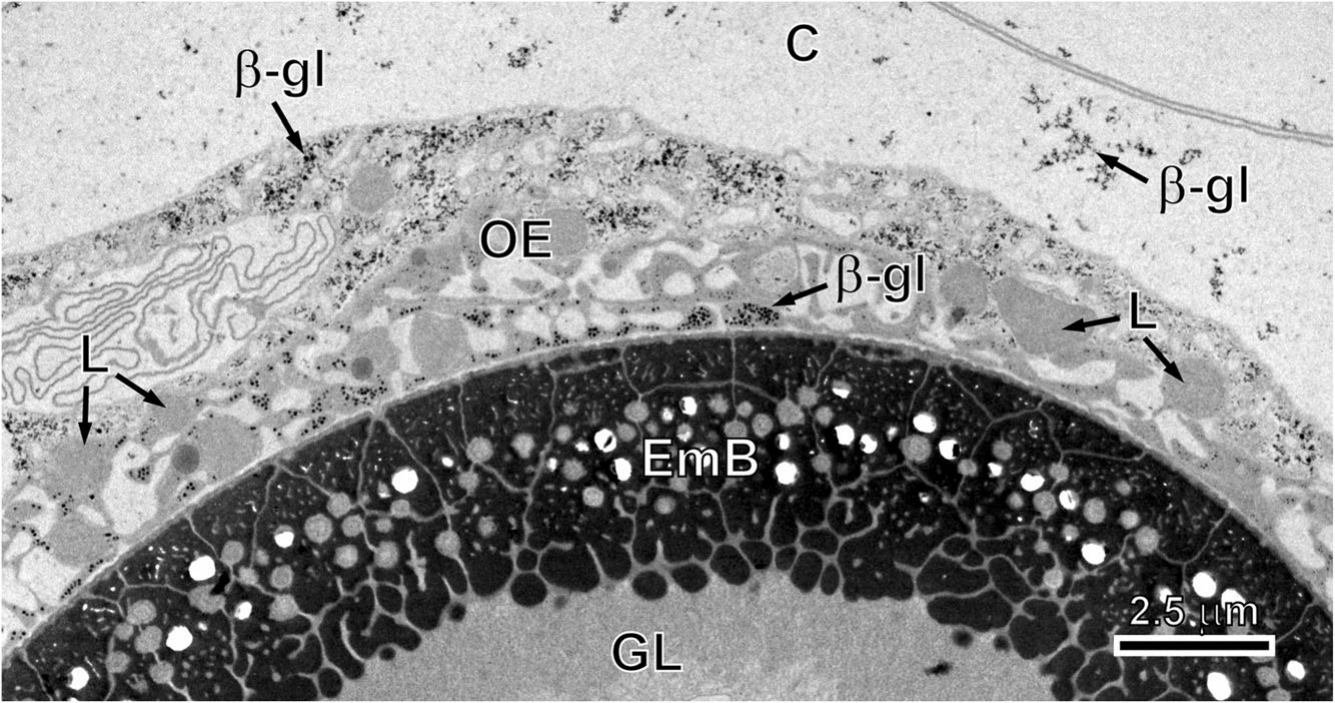
Three external layers of egg envelopes after Thiéry cytochemical test for glycogen. Note (1) several β-glycogen particles and numerous unsaturated lipid droplets in the cytoplasm of the outer envelope, (2) very thick layer of embryophoric blocks in the outer part of the inner envelope with numerous vacuoles and the degenerating small mitochondria embedded between the embryophoric blocks and (3) part of the granular layer of the inner envelope situated under the embryophoric blocks. *β-gl* β-glycogen, *C* vitelline capsule, *EmB* embryophoric blocks of keratin-like protein, *GL* granular layer of embryophore, *L* saturated, osmiophobic lipid droplets, *OE* outer envelope

During advanced phase of embryonic development, the outer and inner envelopes undergo great modifications. The outer envelope remains for a long time as a metabolically active layer that is involved also in the storage of nutritive reserves in the form of glycogen and lipids for final stages of egg development and survival (Fig. 5). The inner envelope is the most important protective layer that, due to a thick layer of embryophoric blocks, assures oncospheral protection and survival (Fig. 5).

The delicate membranous vitelline capsule formed of the material originating from vitelline cells is initially infolded of a thin delicate membrane (Fig. 1a, b) but always remains visible in the intrauterine fully formed eggs at the outer surface of the outer envelope (Fig. 5).

The outer envelope is formed by a fusion of two large macromeres in the preoncospheral phase of the egg formation. At this stage, it is the thickest of all oncospheral envelopes (Figs. 2a, b, 3a and 4a). It consists of a thick layer of syncytial cytoplasm containing large nuclei of two macromeres with prominent electron-dense nucleoli (Figs. 2a, b and 4a). The cytoplasm of the outer envelope contains numerous elongated mitochondria, free ribosomes and polyribosomes randomly dispersed in all cytoplasm (Fig. 3a).

Within the primary inner envelope, two further layers differentiate progressively. They consist of (1) an outer sub-layer involved in embryophore formation (Figs. 2b, 3b and 4a, b) and (2) the inner syncytial sub-layer containing three mesomere nuclei in the early stages (Figs. 2b, 3a, b and 4b) which become very flat, and the entire sub-layer become transformed in granular layer of the inner envelope (Fig. 5).

The beginning of embryophoric block formation can be noted beneath the outer membrane of the inner envelope (Figs. 2b, 3b and 4a). The first keratin-like protein deposits were observed as flattened keratin-like protein islands forming discontinuous layer all around beneath the outer membrane of the inner envelope (Figs. 2b, 3b and 4a). They appear to increase in size by accretion and come to line the plasma membrane without fusing to one another (Figs. 4b and 5).

The embryophore of *E. multilocularis* is thick and composed of numerous keratin-like protein blocks, giving it striated appearance in the sections (Fig. 5).

**Fig. 6:**
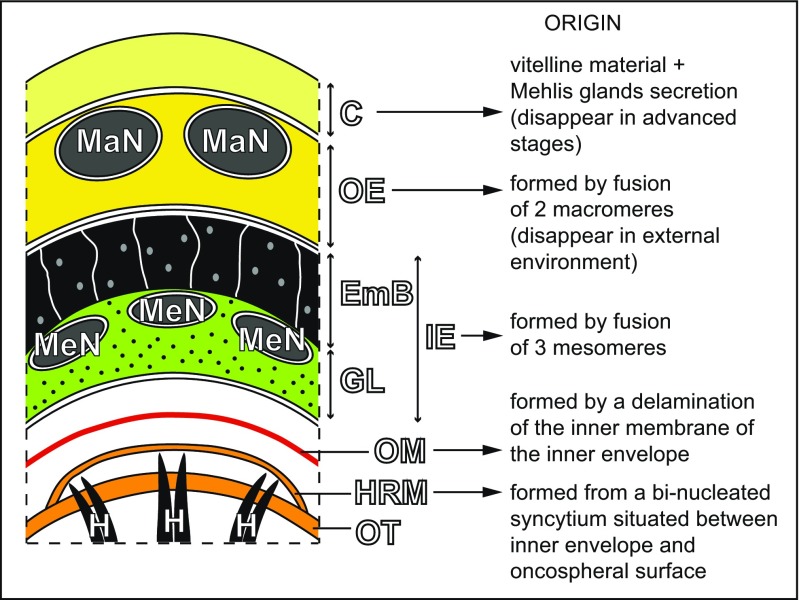
Schematic diagram recapitulating our data on egg envelope ultrastructure with notes on the origin of different layers. *C* vitelline capsule, *EmB* embryophoric blocks of keratin-like protein, *GL* granular layer of embryophore, *H* oncospheral hooks, *HRM* hook region membrane, *IE* inner envelope, *MaN* macromere nucleus, *MeN* mesomere nucleus, *OE* outer envelope, *OM* oncospheral membrane, *OT* oncospheral tegument

*E. multilocularis* eggs are spherical to ellipsoid in shape. In our specimens, mature eggs range in size from 33 to 39 μm and from 26 to 33 μm in their two diameters (36 × 30 μm, average of 15 eggs). The differentiating and mature eggs are surrounded by several layers of four primary envelopes which undergo differentiation into the secondary envelopes, the so-called oncospheral or egg envelopes (Fig. 6).

The embryophore is the principal layer of mature eggs affording physical and physiological protection to the differentiation embryo or oncosphere, since the outer envelope is stripped from the egg before it is liberated. The embryophore is very thick and impermeable, consisting of polygonal blocks composed of an inert keratin-like protein which are held together by a cementing substance. It assures the extreme resistance of eggs enabling them to withstand a wide range of environmental temperatures and physicochemical conditions.

### Functional ultrastructure of egg envelopes

The main function of the vitelline capsule is protection of an early embryo by three types of blastomeres formed during cleavage divisions. At this stage, it contains a small amount of nutritive material in the form of β-glycogen from the degenerating vitellocyte and the uterine wall.

The outer envelope’s function is mainly high metabolic activity from large numbers of mitochondria, ribosomes and polyribosomes and from two large nuclei of macromeres with prominent nucleoli. Storage of nutritive reserves in the form of β-glycogen and highly saturated osmiophobic lipid droplets also represents an important source of energy in the intrauterine eggs. Both the vitelline capsule and outer envelope are stripped from the eggs during their liberation into the external environment.

The inner envelope is the most important protective layer assuring remarkable impermeability and resistance of eggs to a wide range of environmental temperatures, dry climate and a great number of ovicidal substances. However, some impermeability may be due to enzymes that affect the cementing substance of the inert keratin-like protein blocks of the embryophore causing it to disintegrate into its component parts.

In addition to producing keratin-like protein blocks and cement substance, the inner envelope also takes part in the formation of the oncospheral membrane. This innermost membraneous envelope originates by delamination of the inner sub-layer of the inner envelope.

## Discussion

In the study of egg development, the role of Mehlis’ gland or vitellocytes in vitelline capsule or shell formation has been described and confirmed by numerous authors (Leuckart 1886; Smyth and Clegg 1959; Rybicka 1966; Mackiewicz 1968; Świderski et al. 1970). By comparison, our knowledge of egg envelope development and variation in diverse cestodes is less well known.

Over the past 50 years, many papers have been published on the ultrastructure and development of oncospheral envelopes from a wide diversity of cestode species. Indeed, even the more recent literature on cestode egg development and envelopes contains many variations in both the terminology and interpretations. Some of this variation may reflect honest differences of opinion and interpretation. On the other hand, other variations may reflect that cestodes are phylogenetically divers within their respective orders and families and have diverse ecological conditions/cycles that may indeed affect selection for different embryonic or egg envelope structure and that selection and adaptation may vary in different parts of the world.

During the last 50 years, there has been a rapidly growing literature on the ultrastructure of cestode embryonic and egg envelopes of cyclophyllidean cestodes, including taeniids (Pence 1967, 1970; Chew 1983; Conn 1985, 2000; Burt 1987; Conn and Kissel 1991; Świderski et al. 2000, 2001, 2016b; Młocicki et al. 2005; Jabbar et al. 2010a, 2010b). Yet, problems exist.

As indicated by Rybicka (1965, 1966), much confusion and contradiction exist in the terminology used to describe light (LM) and electron microscopical (SEM and TEM) findings of embryonic envelopes of cyclophyllidean cestodes, including taeniids, of medical and veterinary importance. For example, Morseth (1965) reported that the egg envelopes of taeniids are made of eight distinct layers and membranes: (1) egg capsule, (2) vitelline layer, (3) outer embryophoric membrane, (4) embryophore, (5) granular layer, (6) basal membrane of granular layer, (7) oncospheral membrane and (8) limiting membrane. Though Sakamoto (1981) confirmed more or less the results obtained by Morseth (1965) on other taeniids. He wrote in an abstract, however, that the envelopes surrounding *E. multilocularis* oncospheres number up to six: (1) egg capsule, (2) vitelline layer, (3) outer embryophoric membrane, (4) embryophore, (5) granular layer and (6) oncospheral membrane.

Some misinterpretations in Morseth’s paper (Morseth 1965) have been corrected. According to Nieland (1968), the “large cellular structures” in the granular layer of the inner envelopes, situated beneath the embryophore blocks described by Morseth (1965), “might represent the nucleus and nucleolus of this cell.” In reality, the three nuclei situated in this layer certainly represent the nuclei of mesomeres, containing prominent nucleoli, which initially form this inner envelope by their cytoplasmic fusion. We conclude that the “circular bodies” of Morseth (1965) represent mitochondria which in the initial stage of embryophore formation seem to be the focal points for deposition of the block substance. They are certainly involved in the synthesis or maintenance of the integrity of the cement substance that is visible between the blocks. Morseth (1966) described the chemical composition of the blocks as resembling keratin, but the nature of the cement substance is unknown. According to Nieland (1968), some of the impermeability of the eggshell may be due to the cement substance and that the latter is probably the substrate for the enzymes which cause the embryophore to disintegrate into its component blocks. According to Nieland (1968), it is interesting to compare the single layer of blocks of the taeniid embryophores with the embryophore of *Dipylidium caninum* that is composed of two layer of rods at right angles to each other (Pence 1967).

In addition to producing the blocks and cement substance, the inner envelope of taeniid cestodes also appears to participate in the formation of the oncospheral membrane. Rybicka (1966) reported that the oncospheral membrane is formed by the delamination of the innermost part of the inner envelope.

*E. multilocularis* and other taeniid eggs show remarkable impermeability and resistance to the destructive action of ovicidal substances (Rarnell 1965; Mackie and Parnell 1967). Many years earlier, Skvortsov (1942) and Nosik (1952) recognised that the infectivity of taeniid eggs persisted in alcohol or in formalin for several hours. The very high resistance of taeniid eggs against ovicidal agents is certainly dependent upon the thick embryophore blocks of keratin-like protein held together by a cement substance. According to Sakamoto (1981), there is some hope that the impermeable outer embryophoric membrane (i.e. the outer membrane of the inner envelope) may be changed into a porous membrane by digestion of granules packed in the pores. He further concluded that his observations have been used in investigations on ovicidal substances against echinococcal eggs.

Better knowledge of the functional ultrastructure and cytochemistry of egg protecting envelopes in taeniid cestodes may contribute to discovering more effective ovicidal substances against extremely resistance parasite eggs that so far are resistant to tested drugs and physicochemical factors.
